# Use of mechanical circulatory support and survival for heart and heart-kidney transplant recipients in the new allocation system

**DOI:** 10.1016/j.jhlto.2024.100071

**Published:** 2024-02-15

**Authors:** Aurelie Merlo, Hannah F. Bensimhon, Patricia P. Chang, Zhentao Yu, Randall Watkins, Quefeng Li, Mirnela Byku

**Affiliations:** a Department of Surgery, School of Medicine, University of North Carolina, Chapel Hill, North Carolina; b Division of Cardiology, Dartmouth University, Hanover, New Hampshire; c Division of Cardiology, School of Medicine, University of North Carolina, Chapel Hill, North Carolina; d Department of Biostatistics, Gillings School of Global Public Health, University of North Carolina, Chapel Hill, North Carolina

**Keywords:** allocation, heart-kidney transplant, LVAD, MCS, temporary MCS

## Abstract

**Background:**

The 2018 United Network for Organ Sharing organ allocation change aimed to distribute donor hearts to the sickest patients on the waitlist. Whether this change differentially affected outcomes in heart-only vs heart-kidney transplant recipients is unknown.

**Methods:**

This study used the Scientific Registry of Transplant Recipients to compare outcomes, including survival, of heart-only and heart-kidney transplant recipients from 2015 to 2021, from the old vs new allocation system, including use of mechanical circulatory support (MCS), prior to transplant.

**Results:**

During the study period, 16,696 patients underwent heart transplant alone (9,320 in the old and 7,376 in the new system) and 1,156 patients underwent heart-kidney transplant (529 in the old and 627 in the new system). For both heart and heart-kidney transplant populations, there was a 3- to 5-fold increase in the use of temporary MCS. Heart-only recipients had worse survival when temporary MCS was used in the old allocation system. Heart-only recipients with durable MCS had worse survival both in the old and the new allocation system. There was no difference in survival in heart-kidney recipients in the old vs new allocation system, regardless of MCS use.

**Conclusions:**

The new heart allocation system was associated with increased use of temporary MCS in both heart and heart-kidney recipients. However, this change only differentially affected survival in heart-only recipients with improved survival if on temporary MCS, but worse survival if on durable MCS. Unlike prior studies, heart-kidney recipients did not have different outcomes after the heart allocation change, which may reflect outcomes in more current times.

## Background

To maximize the benefit of donor hearts, the United Network for Organ Sharing (UNOS) changed the allocation system in 2018 from a 3-tiered system to a 6-tiered one to allow less variability between groups and better differentiation among clinical status.^1^ The old system resulted in a very large number of patients listed as status 1A, including those with requested exceptions, lumped together with stable mechanical circulatory support (MSC) patients being transplanted during elective 30-day status 1A time. The new organ allocation system attempts to prioritize the highest status to the sickest patients on MCS support.

The impact of the allocation policy change is still being evaluated. Reports demonstrate that certain groups have benefitted—specifically those no longer needing exemptions such as patients with congenital heart disease^2^ or hypertrophic cardiomyopathy.^3^ In addition, it appears that patients who are sicker, as judged by the use of temporary MCS, are transplanted at faster and higher rates,^4^ without an increase in length of stay or early mortality.^5^ Nonetheless, patients who may be more stable, that is, bridge-to-transplant left ventricular assist device (LVAD) patients, are being transplanted at lower rates.^6^, ^7^ The impact of allocation policy change in heart-kidney transplant recipients is not fully understood, but a recent study reported a higher rate of dialysis and 1-year mortality in this cohort.^8^ This current study uses UNOS data from the Scientific Registry of Transplant Recipients (SRTR) to analyze 1-year outcomes, including survival, of heart-only and heart-kidney recipients before and after the change in allocation system, and specifically considers the use of temporary and durable MCS.

## Materials and methods

We analyzed data from the SRTR between January 1, 2015, and December 31, 2021. The study population was stratified into 4 groups with at least 6 months of follow-up time. Subjects were adults (age 18 and older) divided into those receiving heart transplant alone and those receiving combined heart and kidney transplant. Subjects were further divided into those receiving a transplant in the “old” allocation system (transplanted prior to October 18, 2018) and those receiving transplants in the “new” allocation system (transplanted on or after October 18, 2018, through June 30, 2021). Our final study sample was 17,852.

We assessed the use of MCS prior to transplant, including intra-aortic balloon pumps (IABPs), temporary LVADs, right ventricular assist devices, extra-corporeal membrane oxygenation (ECMO), and durable LVADs. For the 4 groups of transplant recipients, descriptive statistics were used to compare baseline characteristics, and the relationship between MCS devices and clinical outcomes at 1 year, including survival, rejection (as defined by UNOS which included both treated and untreated episodes of either antibody-mediated or cellular rejection), need for post-transplant dialysis, and post-transplant length of stay. Since SRTR only has rejection data collected at 1 year, these data were missing for recipients transplanted between July 1, 2021, and December 31, 2021. Survival analysis was performed using Kaplan-Meier analysis and log-rank tests. Cox proportional hazard models were used to adjust for baseline characteristics including age, race, sex, baseline creatinine, and ischemic time of donor organ. Statistical analysis was performed using R software (version 4.1.0).

## Results

Between January 1, 2015, and December 31, 2021, a total of 17,852 patients underwent heart-only or heart-kidney transplant in the United States. Out of the total number of patients, 16,696 patients underwent heart transplantation only and 1,156 patients (6.4%) underwent combined heart-kidney transplant. There were 9,320 heart-only transplants performed in the old allocation system and 7,376 in the new allocation system. There were 529 (5.6%) heart-kidney transplants performed in the old allocation system and 627 (8.5%) heart-kidney transplants in the new allocation system. Table 1 summarizes baseline demographic and clinical data stratified by these 4 subgroups (heart and heart-kidney transplant patients in the old and new allocation system).

**Table 1:** Baseline Characteristics of 16,696 Heart Only and 1,156 Heart-kidney Transplant Recipients, by Old vs New Heart Allocation System (2015-2021)

Demographic and clinical charactereistics	Heart only			Heart-kidney		
	Old (N = 9320)	New (N = 7376)	*p*-value	Old (N = 529)	New (N = 627)	*p*-value
	N (%)	N (%)		N (%)	N (%)	
	Median (IQR)	Median (IQR)		Median (IQR)	Median (IQR)	
Female sex	2,455 (26%)	2,018 (27%)	0.145	84 (16%)	138 (22%)	0.01
Age (year)	57 (17)	57 (17)	0.266	59 (13)	58 (14)	0.23
Race/ethnicity			0.33			0.03
Asian	335 (4%)	260 (4%)		27 (5%)	26 (4%)	
Black	2,091 (22%)	1,722 (23%)		176 (33%)	250 (40%)	
White	5,953 (64%)	4,618 (63%)		273 (52%)	273 (44%)	
Hispanic	827 (9%)	692 (9%)		43 (8%)	62 (10%)	
Other	48 (0.5%)	46 (0.6%)		1 (0.2%)	5 (0.8%)	
Missing	66 (1%)	38 (1%)		9 (2%)	11 (2%)	
Status						
1A (old)	6,251 (67%)	NA	NA	427 (81%)	NA	NA
1B (old)	2,813 (30%)	NA		94 (18%)	NA	
2 (old)	256 (3%)	NA		8 (1.5%)	NA	
1 (new		604 (8%)			70 (11%)	
2 (new)		3,474 (47%)			321 (51%)	
3 (new)		1,446 (20%)			103 (16%)	
4 (new)		1,474 (20%)			75 (12%)	
5 (new)		0 (0%)			58 (9%)	
6( new)		379 (5%)			0 (0%)	
Pretransplant creatinine (mg/dl)	1.2(0.5)	1.1(0.5)	<0.001	2.5(1.8)	2.3(1.8)	0.383
Ischemic time (hours)	3.0(1.4)	3.4 (1.2)	<0.001	2.9(1.2)	3.4(1.2)	<0.001
Any MCS prior to transplant	5,483 (59%)	4,970 (67%)	<0.001	271 (51%)	399 (64%)	<0.001
Durable LVAD prior to transplant	4,541 (49%)	2,190 (30%)	<0.001	183 (35%)	120 (19%)	<0.001
Ventilator prior to transplant	1,820 (20%)	1,132 (15%)	<0.001	109 (21%)	113 (18%)	0.358
Dialysis while on waitlist	153 (2%)	123 (2%)	0.0719	210 (40%)	254 (41%)	0.924
History of sternotomy	6,038 (65%)	3,596 (49%)	<0.001	314 (59%)	274 (44%)	<0.001
Days on waitlist	109 (284)	33 (169)	<0.001	84 (253)	37 (137)	0.001
Donor age (years)	31 (17)	32 (16)	0.002	30 (17)	31 (15)	0.888
Donor female sex	2,820 (30%)	2,091 (28%)	0.008	125 (24%)	167 (27%)	0.27
Donor race/ethnicity			0.498			0.51
Asian	157 (2%)	121 (2%)		7 (1%)	16 (3%)	
Black	1,523 (16%)	1,201 (16%)		72 (14%)	79 (13%)	
White	6,006 (64%)	4,686 (64%)		325 (61%)	389 (62%)	
Hispanic	1,487 (16%)	1,247 (17%)		117 (22%)	133 (21%)	
Other	68 (1%)	61 (1%)		7 (2%)	5 (1%)	
Missing	79 (1%)	60 (1%)		1 (0.2%)	5 (1%)	
Donor BMI (kg/m2)	26.5 (7.4)	26.9 (7.6)	<0.001	26.8 (6.5)	27.2 (7.8)	0.359
Recipient BMI (kg/m2)	27.5 (7.0)	27.6 (7.3)	0.03	27.1 (6.9)	27.5 (6.8)	0.243

BMI, body mass index; IQR, interquartile range; LVAD, left ventricular assist device; MCS, mechanical circulatory support; NA, not available.

Values are expressed as median (interquartile range, IQR) or as number (percentage).

Among heart-only transplant recipients, there was no difference in age, gender, and ethnicity of the recipient between the old and the new allocation systems. However, mean ischemic time was significantly higher in the new allocation system (by 0.4 hours, *p* < 0.001), as was size mismatch (measured by comparison of donor body mass index to recipient body mass index) (Table S1), while days on the waitlist were significantly lower (by 71 days, *p* < 0.001). Patients were more frequently supported with MCS at the time of transplant in the new compared to the old allocation system (15% increase, *p* < 0.001). Patients in the new allocation system were significantly less likely to have a durable LVAD prior to transplant (40% decrease, *p* < 0.001).

Among heart-kidney transplant recipients, a greater proportion of women received a transplant in the new allocation system (38% increase, *p* < 0.001), but the age and ethnicity of recipients did not change with the allocation change. Mean ischemic time was significantly higher in the new allocation system (by 0.4 hours, *p* < 0.001) and waitlist times were significantly shorter (by 66 days, *p* = 0.001). Heart-kidney recipients were more likely to be supported with MCS at the time of transplant in the new compared to the old allocation system (24% increase, *p* < 0.001). They were also significantly less likely to have a durable LVAD prior to transplant in the new allocation system compared to the old (55% decrease, *p* < 0.001).

Table 2 demonstrates the distribution of MCS type for heart-only recipients and heart-kidney recipients. With the new allocation system, there was a significant 3- to 5-fold increase in the use of temporary MCS devices (IABP, Impella, other temporary LVAD, and ECMO) for heart recipients as well as heart-kidney recipients (*p* < 0.001 for all temporary MCS modalities and for both heart and heart-kidney). Overall, this led to a significant decrease in the number of patients transplanted who were supported with a durable LVAD (*p* < 0.001 for both heart-only and heart-kidney).

**Table 2:** Use of Mechanical Circulatory Support Prior to Transplant for Heart Only and Heart-kidney Transplant Recipients, by Old vs New Heart Allocation System (2015-2021)

Type of MCS	Heart only (N = 16,969)			Heart-kidney (N = 1,156)		
	Old (N = 9,320)	New (N = 7,376)	*p*-value	Old (N = 529)	New (N = 627)	*p*-value
No MCS	3,837 (41%)	2,406 (33%)	<0.001	258 (49%)	228 (36%)	<0.001
IABP	692 (7%)	2,087 (28%)	<0.001	53 (10%)	173 (28%)	<0.001
Impella	56 (1%)	367 (5%)	<0.001	8 (2%)	53 (9%)	<0.001
ECMO	75 (1%)	390 (5%)	<0.001	7 (1%)	38 (6%)	<0.001
Temporary LVAD	231 (3%)	521 (7%)	<0.001	37 (7%)	82 (13%)	<0.001
BiVAD	222 (2%)	153 (2%)	0.20	34 (6%)	30 (5%)	0.28
Durable LVAD	4,541 (49%)	2,190 (30%)	<0.001	183 (35%)	120 (19%)	<0.001

BiVAD, biventricular assist device; ECMO, extra-corporeal membrane oxygenation, IABP, intra-aortic balloon pump; LVAD, left ventricular assist device; MCS, mechanical circulatory support.

Table 3 compares outcomes at 1 year for heart-only recipients, stratified by old and new allocation system. For heart-only patients, ECMO patients and temporary LVAD patients had a lower total death rate in the new allocation system (*p* < 0.001). There were also lower rates of rejection at 1 year for patients with any form of MCS (*p* < 0.001) and those specifically supported on IABP (*p* < 0.001) and durable LVADs (*p* = 0.038). Rates of post-transplant dialysis were overall higher in patients without MCS (37% increase, *p* < 0.001), with any form of MCS (13% increase, *p* = 0.003), and with durable MCS (20% increase, *p* = 0.006). For heart-kidney patients, there were no statistically significant differences in any of these post-transplant outcomes comparing old and new allocation systems, even when analyzing by type of MCS (Table S2).

**Table 3:** Six-Month Post-transplant Outcomes by Type of Mechanical Circulatory Support for Heart-Only by Old vs New Heart Allocation System (2015-2021)

Heart-only														
	Total		Death			Rejection			Dialysis			LOS (days)		
	Old	New	Old	New		Old	New		Old	New		Old	New	
	N	N	N (%)	N (%)	*p*-value	N (%)	N (%)	*p*-value	N (%)	N (%)	*p*-value	Med (IQR)	Med (IQR)	*p*-value
No MCS	3,837	2,406	270 (7)	176 (7)	0.715	653 (17)	275 (11)	0.128	366 (10)	313 (13)	<0.001	19 (19)	22 (24)	<0.001
IABP	692	2,087	55 (8)	138 (7)	0.266	154 (22)	245 (12)	<0.001	90 (13)	272 (13)	0.221	22 (22)	22 (21)	0.819
Impella	56	367	6 (11)	27 (7)	0.413	6 (11)	29 (8)	0.595	7 (13)	63 (17)	0.495	19 (12)	21 (18)	0.038
Temp LVAD	231	521	45 (20)	39 (8)	0.001	38 (17)	54 (10)	0.284	50 (22)	82 (16)	0.046	28 (36)	24 (23)	0.126
ECMO	75	390	18 (24)	39 (10)	0.001	15 (20)	50 (13)	0.47	17 (23)	104 (27)	0.623	26 (21)	31 (33)	0.008
BiVAD	222	153	38 (17)	15 (10)	0.065	31 (14)	23 (15)	0.623	44 (20)	28 (18)	0.656	32 (42)	31 (30)	0.771
Durable LVAD	4,541	2,190	383 (8)	219 (10)	0.039	792 (17)	261 (12)	0.038	568 (13)	330 (15)	0.006	23 (25)	25 (29)	0.003

BiVAD, biventricular assist device; ECMO, extra-corporeal membrane oxygenation; IABP, intra-aortic balloon pump; IQR, inter-quartile range; LVAD, left ventricular assist device; MCS, mechanical circulatory support; Med, median; Temp, temporary.

**Figure 1:**
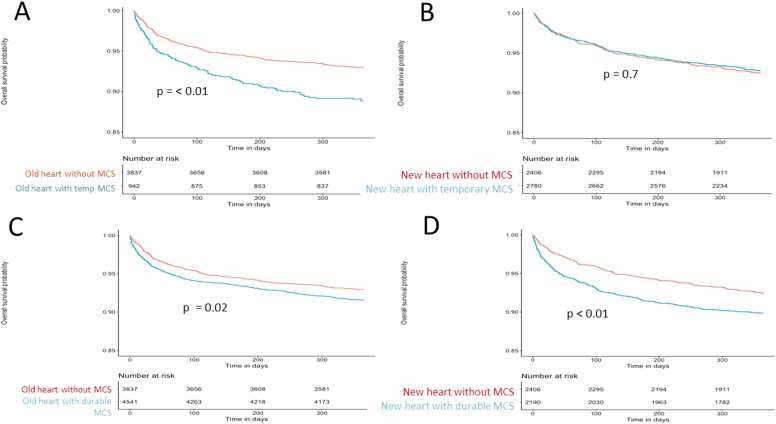
Kaplan-Meier survival curves of heart-only transplant recipients in the old (A) and new (B) allocation era by use of temporary MCS vs no MCS, as well as the heart-only transplant recipients in the old (C) and new (D) allocation era by use of durable MCS vs no MCS. MCS, mechanical circulatory support.

In the old allocation system, MCS of any kind (temporary and durable) was associated with worse survival in heart-only recipients, but this association was not seen in the new system for temporary MCS (Figure 1). There was no difference in survival for heart-kidney recipients based on MCS status in the old or new system (Figure S1). In multivariate analysis, any form of MCS was a predictor of decreased survival in the old system even when accounting for age, gender, race, ischemic time, and creatinine (Table S3a), which remained true for temporary MCS as well (Table S3b). In multivariate analysis, durable MCS was a predictor of worse survival even when accounting for age, race, gender, ischemic time, and creatinine, in both the old and new allocation systems (Tables S3c and S3d).

## Discussion

We performed a retrospective cohort study of all heart-only and heart-kidney transplant recipients from 2015-2021 using the SRTR database. We found that the 2018 change in allocation system resulted in several important changes. We noted decreased time on the waitlist and increased donor ischemic time, for both heart and heart-kidney recipients. This occurred without a significant effect in outcomes for heart-kidney recipients except, however, resulting in a significant increase in overall number of heart-kidney transplants, and, importantly, a higher proportion of female recipients. As reported in prior studies, we found that there was a significant change in use of MCS, with a marked decrease in those transplanted from durable MCS and a significant increase in those transplanted from temporary MCS, which was noted for both heart-only and heart-kidney transplant recipients. While temporary MCS was previously associated with decreased survival in heart-only transplant recipients, we observed no difference between groups in the new allocation system. Importantly, there was no difference in survival in heart-kidney transplant recipients based on temporary MCS use in the old or new system, possibly reflective of increased experience resulting in improved clinical practice and outcomes with temporary MCS over the last few years. Finally, we noted persistently worse survival, both in the old and the new systems for heart-only transplant recipients with durable MCS.

The findings of this study have several important implications. First, they confirm the results from previous studies that have demonstrated reduced waitlist time, similar outcomes in post-transplant survival^5^, ^9^ and rejection,^10^ and higher incidence of post-transplant dialysis.^11^ Secondly, while temporary MCS used to be associated with worse survival post-transplant, this was no longer seen in the new allocation system, encouraging the use of temporary MCS in appropriate patients in the pretransplant setting to achieve optimal clinical stability. Thirdly, heart-kidney transplant recipients with temporary MCS do not show the same trends, suggesting a relative neutral effect of MCS in the heart-kidney group. Lastly, this study demonstrates persistently worse survival in heart-only transplant recipients who are getting transplanted after durable MCS.

One hypothesis as to why patients with temporary MCS have improved survival may be that centers are becoming more adept at using newer technologies, such as ECMO and Impella. Some studies confirm this hypothesis. Agdamag et al showed an increase in the use of temporary MCS from 17.9% to 51.6% with no change in overall survival during their study period.^12^ However, other studies showed that the improvement in outcomes in temporary MCS might be volume-specific. Kim et al showed that preallocation change center volume was not associated with worse outcomes post-temporary MCS; but postallocation change, low volume centers using temporary MCS had worse outcomes.^13^ One specific example of improvements is the increasing use of ambulatory ECMO and Impella. We noted an increase in temporary MCS use in our study population, but not an increase in mechanical ventilation, suggesting temporary MCS patients are ambulatory. Additionally, patients who previously would not have been placed on temporary MCS are now stabilized and optimized with such interventions, which could translate into improved outcomes. Another hypothesis is that some patients may be prematurely or inappropriately placed on temporary MCS, in order to upgrade their status for transplant listing. The notable increase in IABP and Impella use seen in our study may support this theory. In a 2020 study of UNOS Registry heart transplant recipients, short-term survival post-transplant was similar pre and postallocation changes,^14^ perhaps in part because the baseline characteristics of patients pre and postallocation changes are relatively similar, suggesting that we may not be transplanting higher risk patients.^15^ This hypothesis needs further elucidation.

However, the durable MCS group had worse survival when compared to those without durable MCS. This confirms a previous study performed early after the allocation change demonstrating worse survival with durable MCS.^16^ Despite improved post-LVAD long-term survival, potential reasons for worse post-transplant survival in LVAD patients include medical and/or surgical complications associated with LVAD therapy. For a patient to be transplanted after durable LVAD implantation in the new allocation system, they must experience an LVAD complications, such as right ventricular failure, intractable ventricular tachycardia, or persistent infection. The presence of these complications could translate into higher peri-transplant morbidity and a higher risk for complications, such as vasoplegia and end-organ dysfunction, potentially leading to worse post-transplant survival. In addition, the redo sternotomy associated with the transition from durable MCS to transplant contributes to surgical risk. If survival is worse with transplant after durable MCS, one must question if durable MCS in older patients is the right first therapy if it makes transplantation higher risk. While we know that long-term survival after heart transplantation is increased when compared to LVAD therapy, the differences in survival curves are narrowing and may overlap as device technology improves. The best approach is patient-specific and needs to be individualized for each patient with the help of a multidisciplinary team.

Heart-kidney transplant recipients in our analyses had no differences in survival between MCS groups (all MCS, temporary, and durable MCS) and no differences in survival pre and postallocation change. We found this to be puzzling as some studies have shown worse survival in heart-kidney transplant survival postallocation change.^17^ For example, a study by Afflu et al noted similar overall survival pre and postallocation change for heart-kidney recipients, but higher rates of post-transplant dialysis and 1-year mortality in the new allocation system, with the follow-up period only up through 2020.^8^ Another recent study using the SRTR database showed that the policy change may have resulted in worse post-transplant survival in heart-kidney transplant recipients, but their follow-up period was also up through May 2020.^18^ Our study had longer follow-up time, through December 2021, suggesting that the heart-kidney recipients have better outcomes in a more contemporary era. This could potentially be explained by practice changes among transplanting centers, such as modification of patient criteria for listing, as well as increased experience with temporary support strategies over time.

Limitations of this study include limited details on key clinical characteristics, such as the number of previous sternotomies, body surface area to assess size mismatch, the use of organ transport systems, duration of both temporary and durable MCS, type of rejection, and duration of dialysis. Additionally, the heart-kidney patients are a much smaller proportion of the dataset and there were certain variables with high missing data rates, especially when evaluating for rejection. This limited our ability to perform propensity matching between the heart-only and the heart-kidney groups. Nonetheless, this study is based on the national sample of over 17,000 heart transplant recipients in the U.S. and attempts to answer the clinically relevant question of difference in outcomes by use of MCS in heart-only and heart-kidney transplant recipients.

In summary, this retrospective cohort study of heart-only and heart-kidney transplant recipients, pre- and post-2018 allocation change, shows increased use of temporary MCS, improved survival for heart-only recipients on temporary MCS, persistently decreased survival for heart-only transplant recipients on durable MCS, but no difference in outcomes in heart-kidney recipients, which may reflect more current times and practice patterns. More research is needed, to understand why durable MCS patients who undergo heart transplantation are at higher risk and how to mitigate this risk.

## Disclosure statement

The authors declare that they have no known competing financial interests or personal relationships that could have appeared to influence the work reported in this paper.

All authors of the manuscript have no financial relationships to disclose regarding the content herein.

## Appendix A Supplementary material

.

Supplementary Figure 1 Kaplan-Meier survival curves of heart-kidney transplant recipients in the old (A) and new (B) allocation era by use of temporary MCS vs no MCS, as well as the heart-kidney transplant recipients in the old (C) and new (D) allocation era by use of durable MCS vs no MCS
