# Supplementary material for: Use of mechanical circulatory support and survival for heart and heart-kidney transplant recipients in the new allocation system
Source: JHLT Open. 2024 Feb 15;4:100071. doi: 10.1016/j.jhlto.2024.100071 (PMC11935327; doi:10.1016/j.jhlto.2024.100071)
Supplement: Supplementary file 1 — Supplementary material [file mmc1.docx]

Supplemental Material

| Supplemental Table 1. Comparison of recipient and donor BMI (size mismatch) | |
| --- | --- |
|  | P-value |
| Old Heart Only | 0.69 |
| New Heart Only | 0.02 |
| Old Heart Kidney | 0.43 |
| New Heart Kidney | 0.45 |
| *Median recipient BMI was compared to median donor BMI using paired t-test* | |

| Supplemental Table 2. Six-month post-transplant outcomes by type of mechanical circulatory support for heart-kidney recipients by old versus new heart allocation system (2015-2021) | | | | | | | | | | | | | | |
| --- | --- | --- | --- | --- | --- | --- | --- | --- | --- | --- | --- | --- | --- | --- |
| Heart-Kidney | | | | | | | | | | | | | | |
|  | Old | New | Old | New |  | Old | New |  | Old | New |  | Old | New |  |
|  | N | New | N (%) | N (%) | p-value | N (%) | N (%) | p-value | N (%) | N (%) | p-value | Med (IQR) | Med (IQR) | p-value |
| No MCS | 258 | 228 | 20 (8) | 26 (11) | 0.224 | 22 (9) | 5 (2) | 0.067 | 64 (25) | 68 (30) | NA | 26 (29) | 25 (26) | 0.663 |
| Any MCS | 271 | 399 | 29 (11) | 52 (13) | 0.431 | 13 (5) | 21 (5) | 0.168 | 83 (31) | 151 (38) | 0.147 | 34 (42) | 31 (32) | 0.414 |
| IABP | 53 | 173 | 8 (15) | 17 (10) | 0.413 | 3 (6) | 11 (6) | 0.595 | 18 (34) | 60 (35) | 1 | 24 (14) | 30 (24) | 0.038 |
| Impella | 8 | 53 | 0 (0) | 6 (11) | 0.715 | 1 (13) | 3 (6) | 1 | 3 (38) | 20 (38) | 0.033 | 20 (7) | 28 (28) | 0.114 |
| Temp LVAD | 37 | 82 | 2 (5) | 8 (10) | 0.664 | 1 (3) | 4 (5) | 0.554 | 18 (49) | 34 (42) | 0.227 | 49 (58) | 34 (35) | 0.132 |
| ECMO | 7 | 38 | 1 (14) | 12 (32) | 0.636 | 0 (0) | 0 (0) | NA* | 2 (29) | 22 (58) | 0.309 | 37 (17) | 34 (37) | 0.795 |
| BiVAD | 34 | 30 | 3 (9) | 3 (10) | 1 | 1 (3) | 3 (10) | 0.32 | 12 (35) | 15 (50) | 0.351 | 45 (37) | 49 (43) | 0.754 |
| Durable LVAD | 183 | 120 | 19 (10) | 20 (17) | 0.155 | 9 (5) | 6 (5) | 0.568 | 49 (27) | 43(36) | 0.095 | 33 (43) | 31 (38) | 0.709 |
| Abbreviations. BiVAD, biventricular assist device; ECMO, extra-corporeal membrane oxygenation; IABP, intra-aortic balloon pump; IQR, inter-quartile range; LVAD, left ventricular assist device; MCS, mechanical circulatory support; Med, median; NA, not available; Temp, temporary  *Unable to calculate p-value due to insufficient sample size | | | | | | | | | | | | | | |

| Supplemental table 3a. Survival in heart-only transplant recipients with all types of MCS and without MCS in the old allocation system | | |
| --- | --- | --- |
| Parameter | Hazard ratio | p-value |
| No MCS | 0.75 | 0.0001 |
| Age | 1.02 | <0.001 |
| Female Sex | 1.14 | 0.11 |
| Non-Black/non-White race | 1.21 | 0.07 |
| Black race | 0.79 | 0.02 |
| Ischemic time | 1.21 | <0.001 |
| Creatinine | 1.09 | <0.001 |
|  | | |
| Supplemental 3b. Survival in heart-only transplant recipients without and with temporary MCS in the old allocation system | | |
| Parameter | Hazard ratio | p-value |
| No temporary MCS | 0.59 | <0.0001 |
| Age | 1.01 | 0.008 |
| Female Sex | 1.27 | 0.03 |
| Non-Black/non-White race | 1.17 | 0.25 |
| Black race | 0.68 | 0.01 |
| Ischemic time | 1.16 | 0.0008 |
| Creatinine | 1.07 | 0.004 |
|  | | |
| Supplemental 3c. Survival in heart-only transplant recipients without and with durable MCS in the new allocation system | | |
| Parameter | Hazard ratio | p-value |
| Without durable MCS | 0.70 | 0.0006 |
| Age | 1.03 | <0.0001 |
| Female Sex | 1.12 | 0.31 |
| Non-Black/non-White race | 1.32 | 0.06 |
| Black race | 1.01 | 0.95 |
| Ischemic time | 1.13 | 0.001 |
| Creatinine | 1.08 | 0.15 |
|  | | |
| Supplemental 3d. Survival in heart-only transplant recipients without and with durable MCS in the old allocation system | | |
| Parameter | Hazard ratio | p-value |
| No durable MCS | 0.79 | 0.003 |
| Age | 1.01 | <0.0001 |
| Female Sex | 1.10 | 0.32 |
| Non-Black/non-White race | 1.24 | 0.05 |
| Black race | 0.86 | 0.13 |
| Ischemic time | 1.22 | <0.0002 |
| Creatinine | 1.08 | 0.0002 |

Appendix Figure 1. Kaplan-Meier survival curves of heart-kidney transplant recipients in the old (A) and new (B) allocation era by use of temporary MCS vs no MCS, as well as the heart-kidney transplant recipients in the old (C) and new (D) allocation era by use of durable MCS vs no MCS
