# Supplementary figures and images for: Use of mechanical circulatory support and survival for heart and heart-kidney transplant recipients in the new allocation system
Source: JHLT Open. 2024 Feb 15;4:100071. doi: 10.1016/j.jhlto.2024.100071 (PMC11935327; doi:10.1016/j.jhlto.2024.100071)

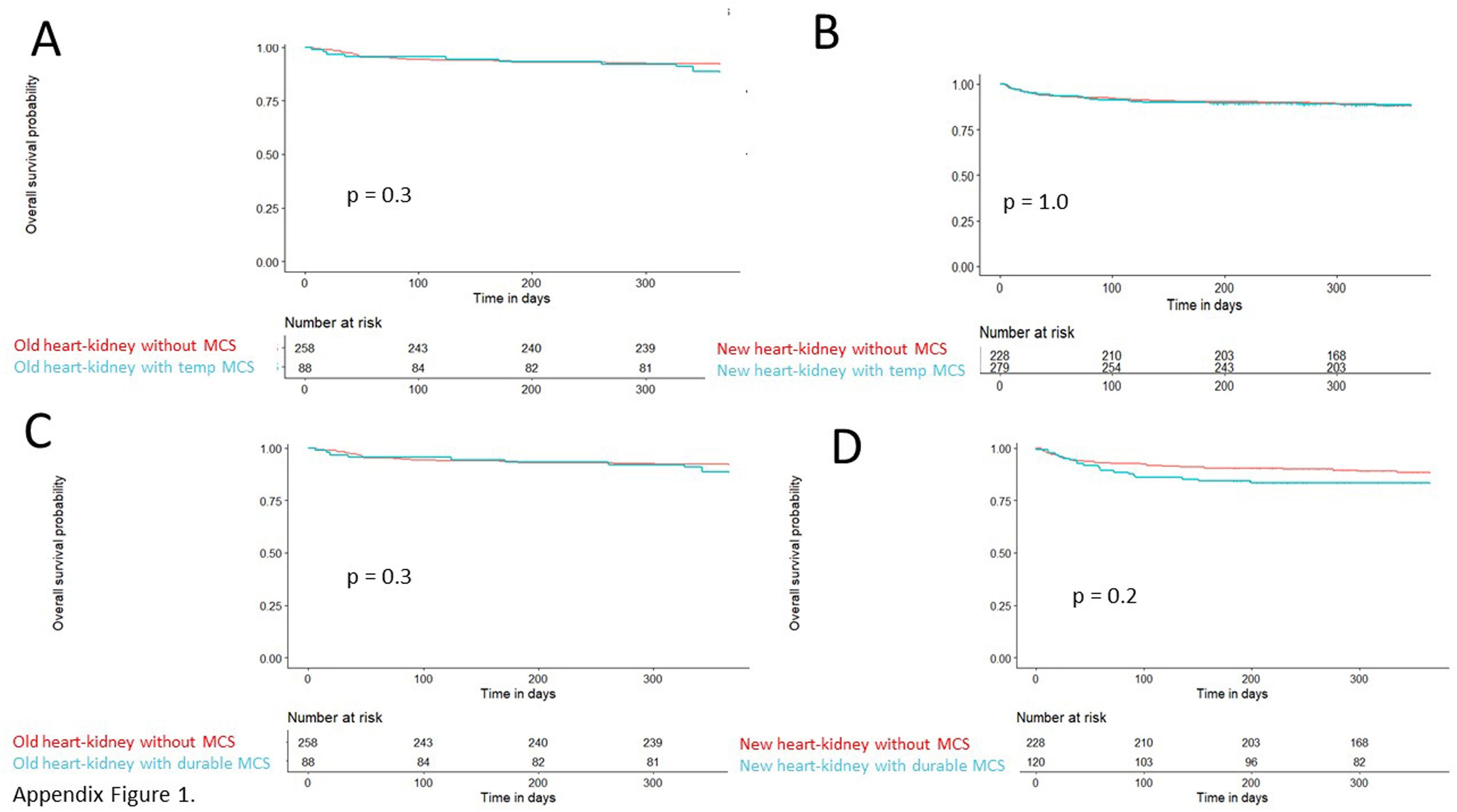

Supplement: Supplementary Figure 1 — Kaplan-Meier survival curves of heart-kidney transplant recipients in the old (A) and new (B) allocation era by use of temporary MCS vs no MCS, as well as the heart-kidney transplant recipients in the old (C) and new (D) allocation era by use of durable MCS vs no MCS [file mmc2.jpg]
